# Supplementary material for: OpenMM 7: Rapid development of high performance algorithms for molecular dynamics
Source: PLoS Comput Biol. 2017 Jul 26;13(7):e1005659. doi: 10.1371/journal.pcbi.1005659 (PMC5549999; doi:10.1371/journal.pcbi.1005659)
Supplement: S1 Source Code — (ZIP) [file pcbi.1005659.s002.zip › OpenMM-7.0.1-Source/docs/api-python/generated/simtk.openmm.app.element.Element.html]

Element — OpenMM 7.0.1.dev-5e86c4f documentation


# Element¶

*class* `simtk.openmm.app.element.``Element`(*number*, *name*, *symbol*, *mass*)¶
:   An Element represents a chemical element.

    The simtk.openmm.app.element module contains objects for all the standard chemical elements,
    such as element.hydrogen or element.carbon. You can also call the static method Element.getBySymbol() to
    look up the Element with a particular chemical symbol.

    Element objects should be considered immutable

    `__init__`(*number*, *name*, *symbol*, *mass*)¶
    :   Create a new element

        |  |  |
        | --- | --- |
        | Parameters: | - **number** (*int*) – The atomic number of the element - **name** (*string*) – The name of the element - **symbol** (*string*) – The chemical symbol of the element - **mass** (*float*) – The atomic mass of the element |

    Methods

    |  |  |
    | --- | --- |
    | `__init__`(number, name, symbol, mass) | Create a new element |
    | `getByAtomicNumber`(atomic\_number) |  |
    | `getByMass`(mass) | Get the element whose mass is CLOSEST to the requested mass. |
    | `getBySymbol`(symbol) | Get the Element with a particular chemical symbol. |

    Attributes

    |  |  |
    | --- | --- |
    | `atomic_number` |  |
    | `mass` |  |
    | `name` |  |
    | `symbol` |  |

    *static* `getBySymbol`(*symbol*)¶
    :   Get the Element with a particular chemical symbol.

    *static* `getByMass`(*mass*)¶
    :   Get the element whose mass is CLOSEST to the requested mass. This method
        should not be used for repartitioned masses

        |  |  |
        | --- | --- |
        | Parameters: | **mass** (*float or Quantity*) – Mass of the atom to find the element for. Units assumed to be daltons if not specified |
        | Returns: | The element whose atomic mass is closest to the input mass |
        | Return type: | Element |

    `__delattr__`¶
    :   x.\_\_delattr\_\_(‘name’) <==> del x.name

    `__format__`()¶
    :   default object formatter

    `__getattribute__`¶
    :   x.\_\_getattribute\_\_(‘name’) <==> x.name

    `__hash__`¶

    `__reduce__`()¶
    :   helper for pickle

    `__reduce_ex__`()¶
    :   helper for pickle

    `__setattr__`¶
    :   x.\_\_setattr\_\_(‘name’, value) <==> x.name = value

    `__sizeof__`() → int¶
    :   size of object in memory, in bytes

High performance molecular simulation on GPUs


### Navigation

- Application Layer
  - Loaders and Setup
  - Representation and Manipulation
  - Simulation
  - Reporting Output
  - Extras
- Library Layer

©2015, Stanford University and the Authors.
|
Powered by Sphinx 1.3.1
& Alabaster 0.7.7
|
Page source
